# V-shaped relationship between platelet-to-lymphocyte ratio and mortality in patients with cardio-cerebrovascular disease: An observational study

**DOI:** 10.1097/MD.0000000000044802

**Published:** 2025-09-26

**Authors:** Jie Li, Yiming Zeng, Chuang Sun, Yue Guan, Mengmeng Shan, Jinzhou Zhu, Qiying Yao, Liang Chen

**Affiliations:** a Department of Cardiology, Ruijin-Hainan Hospital Shanghai Jiao Tong University School of Medicine (Hainan Boao Research Hospital), Qionghai, China; b Department of Cardiology, The Second Affiliated Hospital of Dalian Medical University, Dalian, China; c Basic Medicine College, Dalian Medical University, Dalian, China.

**Keywords:** cardio-cerebrovascular diseases, competing risk model, inflammation, mortality, platelet-to-lymphocyte ratio

## Abstract

This study aimed to elucidate the association between platelet-to-lymphocyte ratio (PLR) and long-term mortality risk in patients with cardio-cerebrovascular diseases (CVD), providing insights for early intervention strategies. Nonlinear associations between the PLR and all-cause mortality were examined using spline smoothing plots analysis. The inflection point of PLR’s effect on all-cause mortality was determined using threshold saturation and log-likelihood ratio tests. Subgroup analyses were performed to assess result stability. A competing risk model was employed to investigate the relationship between PLR and cardiovascular mortality. A total of 5024 adult patients with hypertension were included in this study. The average follow-up duration was 78 months. Spline smoothing plots analysis revealed a V-shaped relationship between PLR and all-cause mortality, with an inflection point at 64.48. When PLR was <64.48, the risk of all-cause mortality declined gradually (HR = 0.979, 95% CI: 0.968–0.990, *P* = .0003) with increasing PLR. Conversely, when PLR was ≥64.48, the risk of all-cause mortality gradually increased (HR = 1.002, 95% CI: 1.001–1.003, *P* < .0001) with increasing PLR. The competing risk model indicated a comparable association between PLR and cardiovascular mortality in patients with CVD. This study establishes a V-shaped PLR-mortality relationship in CVD patients with an inflection point at 64.48. This highlights the potential of PLR as a clinically actionable biomarker for precision risk management, offering a basis for early intervention strategies.

## 1. Introduction

Cardio-cerebrovascular diseases (CVD) encompass heart and brain vascular disorders, including coronary heart disease, stroke, myocardial infarction, and angina.^[1]^ Pathogenesis begins with lipid oxidation in compromised endothelium, triggering macrophages to engulf oxidized lipids and secrete pro-inflammatory mediators and growth factors. This cascade promotes smooth muscle cell proliferation, leading to characteristic atherosclerotic fatty-fibrous lesions.^[2]^ As a leading global non-communicable disease, CVD accounts for approximately one-third of worldwide mortality.^[3]^ Driven by population growth and aging, its burden reached 688 million cases and 19.8 million deaths by 2022.^[4]^ Despite significant prevention and management advances, CVD remains a major contributor to global morbidity, mortality, and rising healthcare costs.^[5]^ Early adverse event risk assessment enables targeted interventions, improving prognosis and potentially curbing global CVD incidence.

While essential for tissue repair, uncontrolled chronic inflammation causes significant damage.^[6]^ In CVD, inflammation is present early-stage. Endothelial damage triggers local responses, inducing systemic low-grade inflammation. Increased metabolic risks exacerbate cardiovascular inflammation, culminating in adverse events and mortality.^[7–9]^ The platelet-to-lymphocyte ratio (PLR), calculated as platelet count divided by lymphocyte count, is a potential indicator of inflammation and thrombosis.^[10]^ PLR reflects inflammatory status and is linked to adverse prognoses in various diseases.^[11–13]^ Recently, PLR has shown predictive value for cardiovascular events in CVD patients, including acute myocardial infarction^[14]^ and cardioembolic stroke.^[15]^

Although prior research established associations between PLR and post-event prognosis in non-ST elevation myocardial infarction,^[16]^ the nature of PLR’s relationship with long-term mortality across diverse CVD populations remains undefined. This study hypothesizes a nonlinear association with threshold effects between PLR and mortality in CVD, reflecting a dual-directional pathological mechanism: immunodeficiency risk at low PLR versus thrombotic inflammation at high PLR. The findings aim to provide clinicians with novel risk assessment tools and evidence to strengthen early intervention strategies.

## 2. Materials and methods

### 2.1. Study population

This study used data from the National Health and Nutrition Examination Survey (NHANES) database, accessible through the official website (https://wwwn.cdc.gov/nchs/nhanes/Default.aspx). All experiments involved in this paper were performed in accordance with relevant guidelines and regulations.

**Figure 1.**
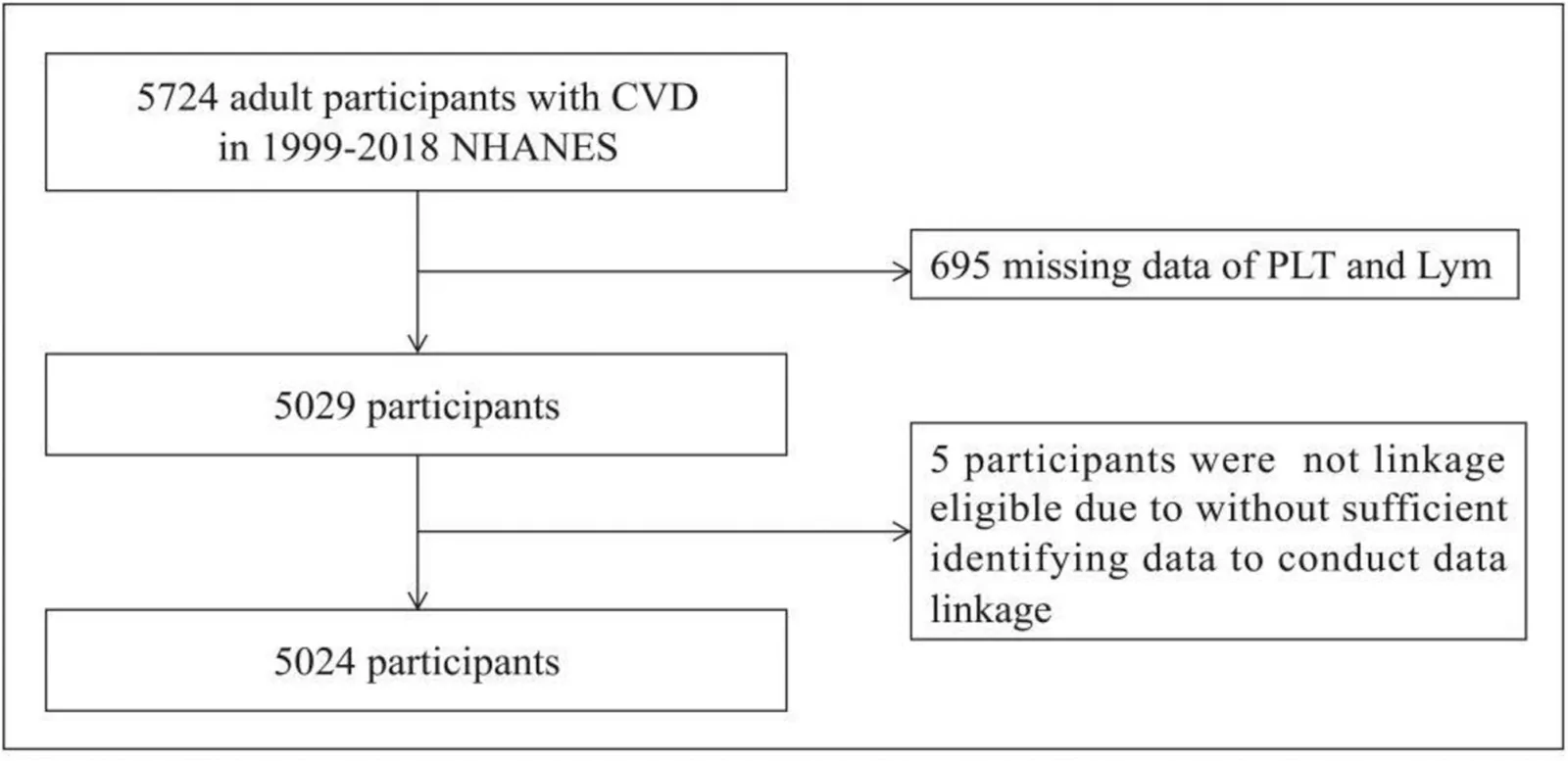
Participant inclusion flowchart. CVD = cardio-cerebrovascular diseases, NHANES = National Health and Nutrition Examination Survey.

Between 1999 and 2018, a total of 5724 adult participants diagnosed with CVD, from diverse racial backgrounds and multiple regions were included in the NHANES database. Participants were categorized as having CVD if they responded “Yes” to any of the 4 questions regarding prior diagnoses of coronary heart disease, angina, myocardial infarction, or stroke. Among these, 695 participants were excluded due to missing platelet and lymphocyte count data. Additionally, 5 participants lacked sufficient information to link with the National Death Index data, resulting in missing follow-up outcomes regarding survival status and their subsequent exclusion. Consequently, a total of 5024 participants were included in this study (Fig. 1).

### 2.2. Ethical statement

The study protocol received approval from the Ethics Review Committee of the National Center for Health Statistics (NCHS) in the United States, and all participant provided informed consent. All experiments involved in this paper are performed in accordance with relevant guidelines and regulations.

### 2.3. Survival status and follow-up duration

The NCHS correlates NHANES data from various surveys with death certificate records in the National Death Index. This study pre-specified 2 primary outcomes: all-cause mortality and cardiovascular-specific mortality. Cardiovascular mortality was defined according to the codebook of NCHS, which divided the causes of death into 10 categories, including “Diseases of heart” and so on.

### 2.4. Baseline characteristics

The baseline characteristics of the participants were collected through computer-assisted personal interviews, questionnaire surveys, and blood tests. These data encompassed gender, age, race, education level, ratio of family income to poverty, body mass index (BMI), smoking history, alcohol consumption history, history of hypertension, hypercholesterolemia, diabetes, or heart failure, as well as alanine aminotransferase (ALT), aspartate aminotransferase (AST), albumin, globulin, creatinine, uric acid, platelet count, lymphocyte count, white blood cell count (WBC), monocyte, and neutrophil. Smokers were defined as individuals who had smoked at least 100 cigarettes in their lifetime, while drinkers were defined as those who had consumed at least 12 alcoholic beverages in the past 12 months. The presence of hypertension, hypercholesterolemia, diabetes, heart failure, and stroke was based on participants’ self-reported diagnoses by a physician. PLR was calculated as the ratio of platelet count to lymphocyte count, represented as a continuous variable.

### 2.5. Statistical analysis

Categorical variables are presented as counts (%), while continuous variables are expressed as medians with first and third quartiles. Differences in categorical variables between the survival and death groups were compared using the chi-square test, and differences in continuous variables between groups were compared using the rank sum test. Spline smoothing plot analysis based on a restricted cubic spline function was used to explore the potential linear relationship between PLR and all-cause mortality risk. The inflection point of PLR’s impact on all-cause mortality risk was determined through threshold saturation and log-likelihood ratio tests. Subsequently, result stability was confirmed through subgroup analysis and interaction testing. Furthermore, the association between PLR and cardiovascular mortality risk was validated using a competing risk model. Before regression analysis, all covariates underwent collinearity screening, with none exhibiting a variance inflation factor exceeding 5. Three regression models were established to adjust for confounding factors systematically. Model 1 remained unadjusted for confounders, while model 2 included adjustments for age, gender, and race. In model 3, in addition to the fixed demographic adjustments, further covariates were selected based on the principle of covariate screening. Specifically, a confounding factor was included in the regression model if the change in *P*-values exceeded 10% when comparing its value before and after its introduction into the model. Ultimately, the adjustment factors in model 3 comprised age, gender, race, education level, the ratio of family income to poverty, BMI, smoking, alcohol consumption, ALT, albumin, globulin, creatinine, uric acid, hypertension, hypercholesterolemia, diabetes, and heart failure. Additionally, receiver operating characteristic curves to assess the predictive value of PLR.

In this study, data analysis was conducted using EmpowerStats and Stata software. *P* < .05 was considered statistically significant.

## 3. Results

### 3.1. Baseline characteristics

This study enrolled 5024 adults with CVD, who were followed up for an average of 78 months. Among them, 2800 patients survived, while 2224 patients died during the follow-up period. Compared to the surviving patients, deceased patients were older and exhibited lower ALT, platelet, and lymphocyte levels but higher globulin, creatinine, and uric acid levels. They were also more likely to be male, alcohol-dependent, hypertensive, and diabetic. Differences in race, education level, ratio of family income to poverty, BMI, and prevalence of hypercholesterolemia were also observed. Detailed results are presented in Table 1.

**Table 1:** Basic characteristics of participants.

	All	Survival	All-cause mortality	*P*
	5024	2800	2224	
Time, mo	78 (39−130)	91 (45−146)	67 (33−109)	<.001
Age, yr	69 (60−78)	64 (55−72)	76 (67−80)	<.001
Gender				<.001
Male	2888 (57.48)	1535 (54.82)	1353 (60.84)	
Female	2136 (42.52)	1265 (45.18)	871 (39.16)	
Race				<.001
Mexican American	592 (11.78)	362 (12.93)	230 (10.34)	
Other Hispanic	307 (6.11)	233 (8.32)	74 (3.33)	
Non-Hispanic White	2846 (56.65)	1351 (48.25)	1495 (67.22)	
Non-Hispanic Black	980 (19.51)	619 (22.11)	361 (16.23)	
Other race	299 (5.95)	235 (8.39)	64 (2.88)	
Education level				<.001
<High school	1798 (35.79)	882 (31.50)	916 (41.19)	
High school graduate or general equivalency diploma	1240 (24.68)	686 (24.50)	554 (24.91)	
>High school	1974 (39.29)	1228 (43.86)	746 (33.54)	
Unknown	12 (0.24)	4 (0.14)	8 (0.36)	
Ratio of family income to poverty				<.001
≤1	1048 (20.86)	631 (22.54)	417 (18.75)	
Between 1 and 3	2304 (45.86)	1151 (41.11)	1153 (51.84)	
>3	1247 (24.82)	778 (27.79)	469 (21.09)	
Unknown	425 (8.46)	240 (8.57)	185 (8.32)	
BMI				<.001
<30 kg/m^2^	2509 (49.89)	1366 (48.79)	1142 (51.35)	
≧30 kg/m^2^	1878 (37.34)	1225 (43.75)	651 (29.27)	
Unknown	642 (12.77)	209 (7.46)	431 (19.38)	
Smoking				.583
No	2084 (41.48)	1171 (41.82)	913 (41.05)	
Yes	2940 (58.52)	1629 (58.18)	1311 (58.95)	
Alcohol use				.01
No	1959 (38.99)	1136 (40.57)	823 (37.01)	
Yes	3065 (61.01)	1664 (59.43)	1401 (62.99)	
Hypertension				.071
No	1420 (28.26)	820 (29.29)	600 (26.98)	
Yes	3604 (71.74)	1980 (70.71)	1624 (73.02)	
Hypercholesterolemia				<.001
No	2103 (41.86)	1080 (38.57)	1023 (46.00)	
Yes	2921 (58.14)	1720 (61.43)	1201 (54.00)	
Diabetes				.040
No	3462 (68.91)	1963 (70.11)	1499 (67.40)	
Yes	1562 (31.09)	837 (29.89)	725 (32.60)	
Heart failure				<.001
No	3914 (77.91)	2312 (82.57)	1602 (72.03)	
Yes	1110 (22.09)	488 (17.43)	622 (27.97)	
ALT, U/L	23.00 (17.00−39.00)	26.00 (18.00−53.00)	20.00 (15.00−29.00)	<.001
AST, U/L	23.00 (19.00−27.00)	23.00 (19.00−27.00)	23.00 (19.00−27.00)	.364
Albumin, g/L	41.00 (39.00−44.00)	41.00 (40.00−44.00)	41.00 (39.00−43.00)	<.001
Globulin, g/L	30.00 (27.00−33.00)	29.00 (26.00−32.00)	30.00 (27.00−34.00)	<.001
Creatinine, µmol/L	88.40 (70.70−106.10)	83.10 (69.00−98.40)	97.20 (79.60−114.90)	<.001
Uric acid, µmol/L	350.90 (291.50–410.40)	339.00 (279.60–398.50)	354.90 (297.40–434.20)	<.001
WBC, 10^3^/UL	7.10 (5.90–8.60)	7.10 (5.90−8.50)	7.20 (5.90–8.80)	.010
Monocyte, 10^3^/UL	0.60 (0.50–0.70)	0.60 (0.50–0.70)	0.60 (0.50–0.70)	<.001
Neutrophil, 10^3^/UL	4.30 (3.30–5.40)	4.10 (3.20–5.20)	4.40 (3.50–5.60)	<.001
Platelet, 10^3^/UL	226.00 (187.00–273.00)	228.00 (189.00–273.00)	225.00 (185.00–274.00)	.251
Lymphocyte, 10^3^/UL	1.90 (1.50−2.40)	2.00 (1.60−2.50)	1.80 (1.30−2.30)	<.001
PLR	120.00 (92.61−157.65)	114.79 (88.87−147.50)	127.62 (97.08−172.54)	<.001

ALT = alanine aminotransferase, AST = aspartate aminotransferase, BMI = body mass index, PLR = platelet-to-lymphocyte ratio, WBC = white blood cell.

### 3.2. Relationship between PLR and long-term all-cause mortality risk in patients with CVD

**Figure 2.**
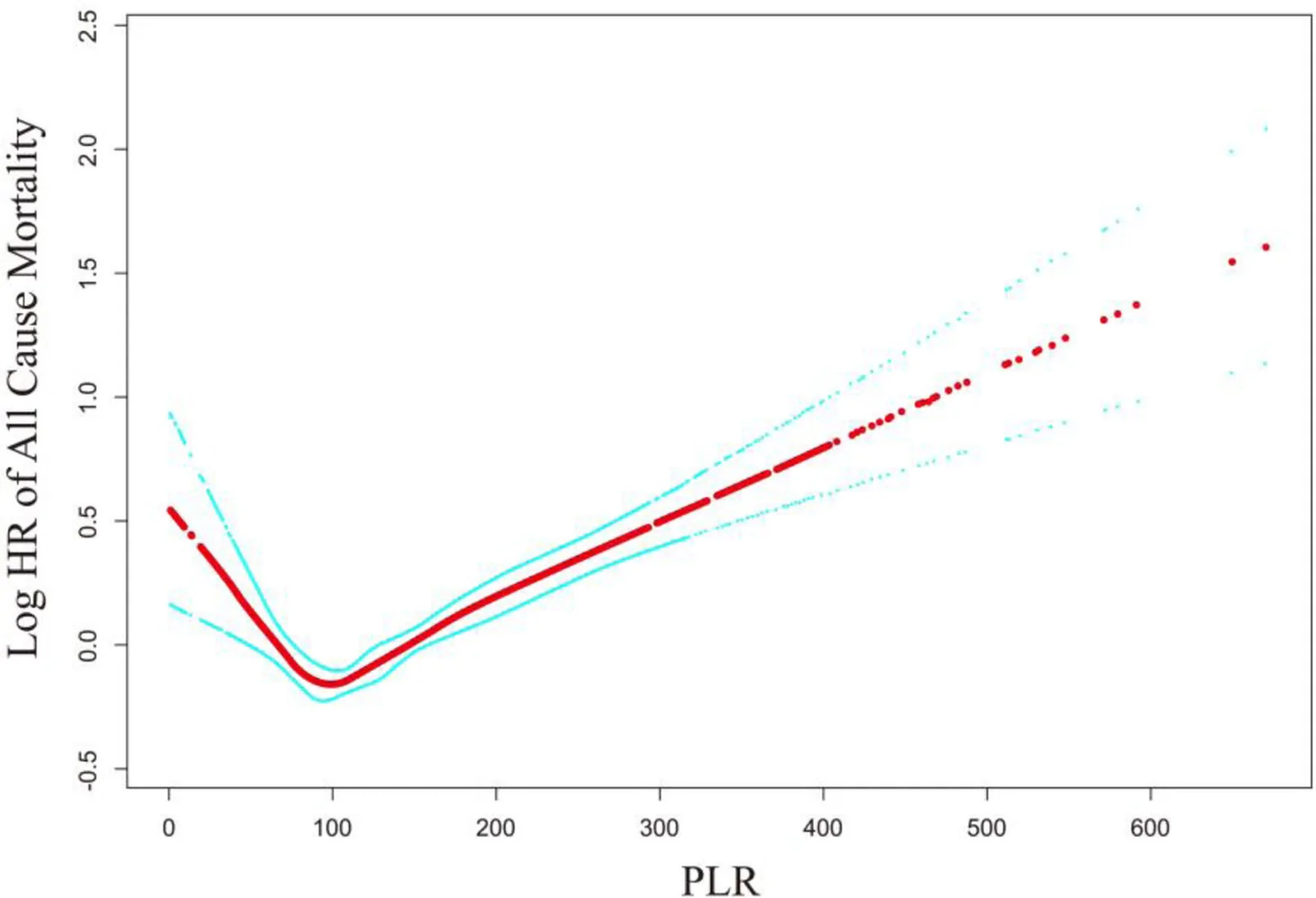
Spline interpolation between PLR and all-cause mortality risk in patients with CVD. CVD = cardio-cerebrovascular diseases, HR = hazard ratio, PLR = platelet-to-lymphocyte ratio.

Spline smoothing analysis revealed a V-shaped relationship between PLR and all-cause mortality risk in CVD patients, with a distinct inflection point (Fig. 2). Threshold saturation analysis further confirmed the nonlinear correlation’s statistical significance. When PLR < 64.48, mortality risk decreased with rising PLR (HR = 0.977, 95% CI: 0.968–0.985, *P* < .0001). Conversely, risk increased progressively with higher PLR when PLR ≥ 64.48 (HR = 1.002, 95% CI: 1.001–1.003, *P* < .0001). Detailed information is presented in Table 2.

**Table 2:** Threshold saturation analysis between PLR and all-cause mortality risk in patients with CVD.

	Model 1	Model 2	Model 3
	HR (95% CI) *P*	HR (95% CI) *P*	HR (95% CI) *P*
PLR < 64.48	0.978 (0.971, 0.986)	0.981 (0.974, 0.989)	0.979 (0.968, 0.990)
	<.0001	<.0001	.0003
PLR ≧ 64.48	1.003 (1.002, 1.004)	1.002 (1.001, 1.003)	1.002 (1.001, 1.003)
	<.0001	<.0001	<.0001
*P* for Logarithmic likelihood ratio test	<.0001	<.0001	<.0001

Model 1: No covariate adjustments were made.

Model 2: Adjustments were made for age, gender, and race.

Model 3: Adjustments were made for age, gender, race, education level, ratio of family income to poverty, BMI, smoking, alcohol consumption, ALT, albumin, globulin, creatinine, uric acid, WBC, monocyte, neutrophil, hypertension, hypercholesterolemia, diabetes, and heart failure.

ALT = alanine aminotransferase, BMI = body mass index, CI = confidence interval, CVD = cardio-cerebrovascular diseases, HR = hazard ratio, PLR = platelet-to-lymphocyte ratio, WBC = white blood cell.

### 3.3. Subgroup analysis and interaction testing

**Figure 3.**
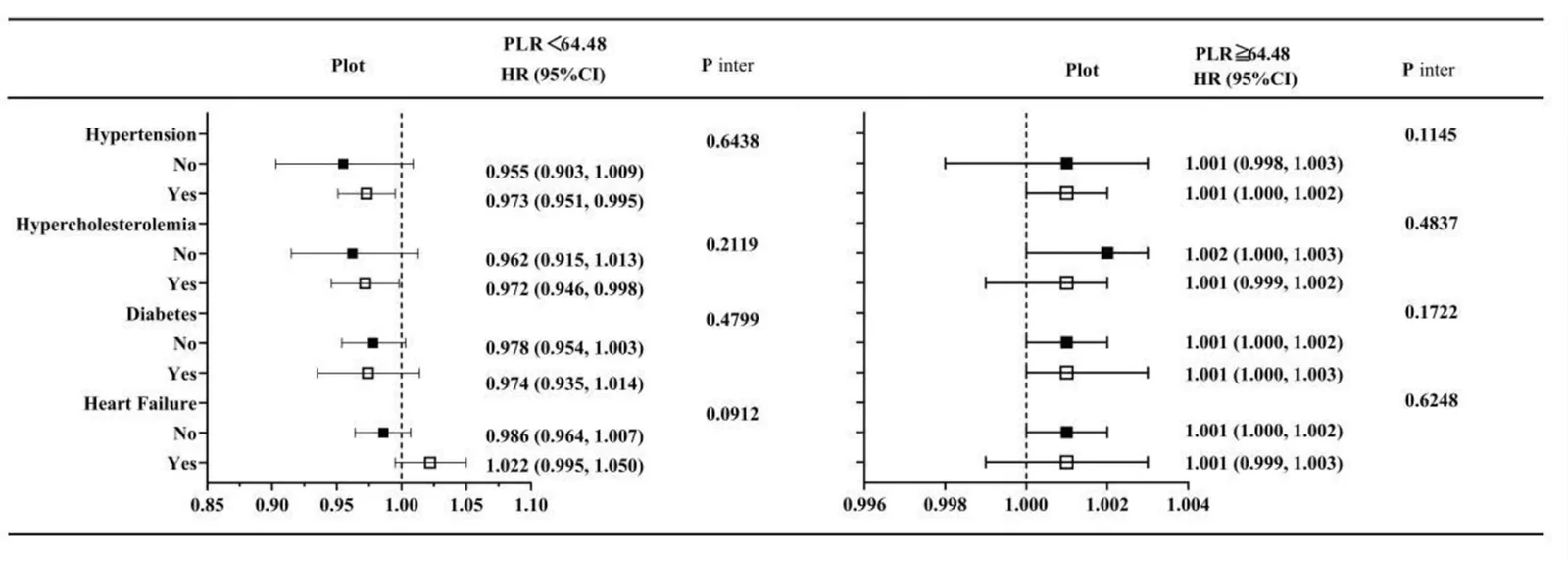
Subgroup analysis and interaction testing. Adjustments were made for age, gender, race, education level, ratio of family income to poverty, BMI, smoking, alcohol consumption, ALT, albumin, globulin, creatinine, uric acid, WBC, monocyte, neutrophil, hypertension, hypercholesterolemia, diabetes, and heart failure (excluding stratification factors). ALT = alanine aminotransferase, BMI = body mass index, CI = confidence interval, HR = hazard ratio, PLR = platelet-to-lymphocyte ratio, WBC = white blood cell.

No significant effect modifiers were identified in subgroup analysis and interaction testing, as shown in Figure 3.

### 3.4. Relationship between PLR and cardiovascular mortality risk in patients with CVD

The relationship between PLR and cardiovascular mortality risk in patients with CVD was examined using a competing risk model. The findings showed that CVD mortality risk decreased with rising PLR at values <64.48 and increased at PLR ≥ 64.48, although the *P*-value did not indicate statistically significant results. Detailed results are presented in Table 3.

**Table 3:** Competing risk model for the relationship between PLR and CVD mortality.

	PLR < 64.48	PLR ≧ 64.48
	HR (95% CI) *P*	HR (95% CI) *P*
Death due to CVD	0.981 (0.947, 1.017) .300	1.001 (0.999, 1.002) .664
All other causes of death	0.988 (0.962, 1.014) .370	1.002 (1.001, 1.002) .046

Adjustments were made for age, gender, race, education level, ratio of family income to poverty, BMI, smoking, alcohol consumption, ALT, albumin, globulin, creatinine, uric acid, WBC, monocyte, neutrophil, hypertension, hypercholesterolemia, diabetes, and heart failure.

ALT = alanine aminotransferase, BMI = body mass index, CI = confidence interval, CVD = cardio-cerebrovascular diseases, HR = hazard ratio, PLR = platelet-to-lymphocyte ratio, WBC = white blood cell.

### 3.5. Predictive value of PLR

**Figure 4.**
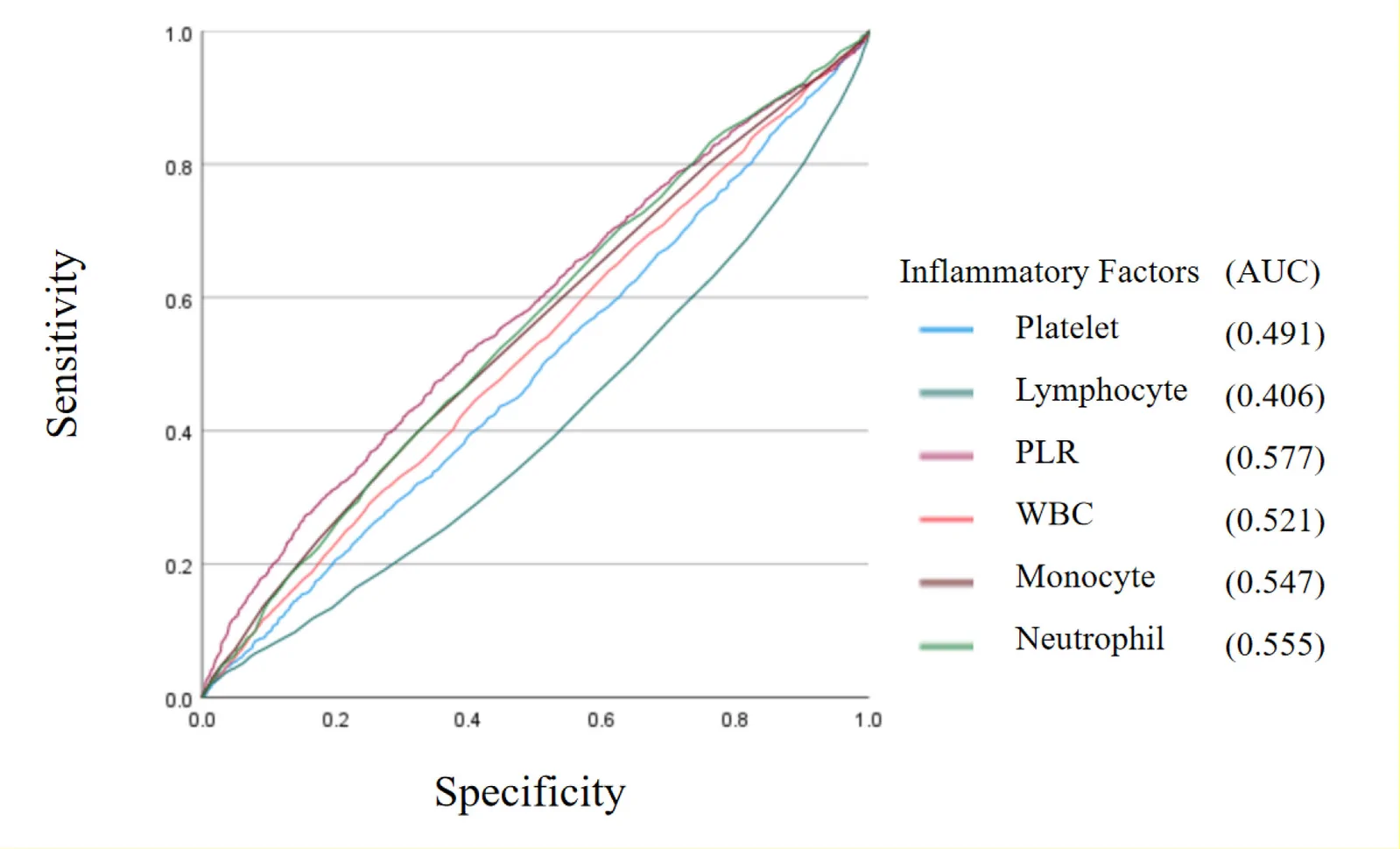
ROC curve. AUC = area under curve, PLR = platelet-to-lymphocyte ratio, ROC = receiver operating characteristic, WBC = white blood cell.

In the receiver operating characteristic curve analysis, PLR demonstrated a higher area under curve than other inflammatory factor (Fig. 4).

## 4. Discussion

This study, using the NHANES database, revealed a V-shaped relationship between PLR and long-term all-cause mortality risk in patients with CVD. Specifically, as PLR increased, the mortality rate initially decreased before rising again among patients with CVD, with the inflection point at 64.48. Similar trends were observed between PLR and cardiovascular mortality rates.

The PLR reflects platelet and lymphocyte balance and is considered a more stable, accessible marker than other leukocyte subtypes or inflammatory indicators.^[10]^ Initially used in cancer prognostics,^[17,18]^ PLR has emerged as a dual marker of inflammation and thrombosis in cardiovascular diseases.^[19]^ While multiple studies have reported linear associations between elevated PLR and adverse outcomes in acute coronary syndromes,^[20–23]^ the novel V-shaped relationship identified in our general CVD population suggests more complex pathophysiology. This divergence likely reflects differences in study populations and disease stages-previous work focused on acute events where extreme thrombotic/inflammatory responses dominate,^[14]^ whereas our cohort included chronic CVD where both deficient and excessive responses may be detrimental. A standardized diagnostic threshold for PLR remains undefined.^[24]^ Notably, 2 recent studies support our threshold findings: Tamaki et al identified PLR ≥ 193 as predictive in heart failure,^[25]^ while Zhang et al found PLR ≥ 107.57 significant in hemodialysis patients.^[26]^ These varying thresholds, including our 64.48 cutoff, likely represent population-specific optima between pro-thrombotic and immunosuppressive risks. Threshold variability likely reflects population differences, this NHANES-based study utilized larger, ethnically diverse cohorts, enhancing reliability. Both low and high PLR signify risk, their fluctuations mirror the body’s inflammatory status. Elevated PLR reflects increased platelet activity or lymphopenia, marking inflammation and thrombosis risk.^[27,28]^ Conversely, low PLR may indicate immune dysfunction, such as thrombocytopenia or immature platelets causing immunodeficiency.^[29]^ Inflammatory states can conversely increase lymphocytes, lowering PLR.^[30]^

The V-shaped PLR-mortality relationship carries considerable clinical implications. Clinically, PLR monitoring offers a practical risk-stratification tool for cardiology practice: patients with PLR < 64.48 may require immunodeficiency or bone marrow evaluation given impaired vascular repair capacity,^[29]^ while those with PLR ≥ 64.48 warrant intensified anti-inflammatory/antiplatelet regimens due to progressive thrombosis risk.^[19]^ Therapeutically, the inflection point establishes a target range where serial PLR measurements could guide personalized treatment titration. This risk-adapted approach aligns with precision medicine paradigms in CVD management.^[31]^

The inflammatory process is believed to play a pivotal role in the development and complications of CVD,^[32]^ contributing to pathological mechanisms such as endothelial dysfunction, thrombus formation, and atherosclerosis. Consequently, it heightens the risk of various cardiovascular events, including coronary heart disease, heart failure, and hypertension.^[31]^ Additionally, inflammatory mediators can induce apoptosis of myocardial cells, leading to disturbances in cardiac electrophysiology and structural remodeling via fibroblast activation, thereby exacerbating inflammation.^[33,34]^ Within the inflammatory response, diverse inflammatory cells and molecules are activated and interact, forming a complex network that ultimately leads to vascular damage and cardiovascular disease progression.^[35]^ While early stages are often clinically asymptomatic, acute and chronic inflammation can progress to active episodes triggered by inflammatory mediators released from sites of vascular injury, infection, or subclinical plaque disruption.^[9]^

PLR monitoring could serve as a practical tool for risk stratification in outpatient cardiology practice. PLR integrates platelet and lymphocyte counts, both pivotal in CVD pathophysiology. Platelets drive inflammation^[36]^ while maintaining hemostasis and endothelial integrity.^[37]^ Intact endothelium prevents platelet contact, but vascular injury triggers platelet adhesion to subendothelial matrix for hemostasis and healing.^[38]^ Activated platelets bind leukocytes/endothelium, promoting monocyte-to-macrophage conversion and releasing inflammatory mediators that induce megakaryocyte proliferation and reactive thrombocytosis.^[39,40]^ Under pathological stimuli (hypertension, dyslipidemia, inflammation), these cells accumulate in arterial intima, accelerating CVD.^[41]^ In CVD patients, activated platelets aggregate on damaged vessels, forming occlusive clots that cause myocardial infarction/stroke,^[42,43]^ while releasing inflammatory/growth factors that exacerbate vascular damage.^[44]^

Lymphocytes, key immune cells, participate throughout inflammation. T lymphocytes critically influence CVD development.^[45]^ Activated lymphocytes release interleukins, interferons, and TNF, exacerbating endothelial damage and thrombosis to drive CVD progression.^[46]^ Activated endothelial cells release factors attracting lymphocytes, which infiltrate arterial walls, initiating inflammation. Subsequent cell/cytokine accumulation oxidizes and deposits lipids, causing atherosclerosis.^[47]^ Abnormal lymphocyte activation may further trigger autoimmune cardiovascular damage.^[48]^ Systemic inflammation inversely correlates with lymphocyte counts,^[49]^ aligning with this study’s finding of lower counts in deceased CVD patients versus survivors. Reduced lymphocytes may thus indicate poor CVD outcomes. The PLR ratio concurrently reflects thrombosis risk and inflammatory status.

This study establishes PLR as a novel predictor of adverse CVD outcomes. PLR correlates with both all-cause and cardiovascular mortality, suggesting its utility as a prognostic marker for early intervention. For patients with PLR < 64.48, immune support could be prioritized, whereas those with PLR ≥ 64.48 may benefit from anti-inflammatory or antiplatelet therapies.

### 4.1. Limitation

This study had a few limitations. First, the study population was derived from NHANES and comprised participants solely from the United States. Consequently, the results may not be directly applicable to populations in other countries. Clinicians should exercise caution when using PLR to evaluate prognosis in patients with CVD from other countries. Further validation of the results may be achieved by exploring the relationship between CVD progression and PLR in other multicenter cohorts. Second, lymphocyte and platelet levels in the body fluctuate, and results from a single measurement may not accurately reflect patients’ long-term physiological status. Finally, the definition of coronary heart disease in this study relied on self-reported conditions, including angina, myocardial infarction, and stroke. Nonetheless, early-stage CVD often lacks evident symptoms, potentially leading to missed diagnoses in many patients due to irregular medical checkups, thereby increasing the risk of type II errors in this study.

## 5. Conclusion

This study establishes a significant V-shaped association between PLR and mortality risk in CVD patients, with an inflection point at 64.48. These findings position PLR as a clinically actionable prognostic biomarker for early risk stratification. Maintaining PLR near this threshold may facilitate dynamic risk monitoring and enable proactive clinical management to attenuate CVD-related mortality.

## Acknowledgments

We would like to thank Editage (www.editage.cn) and DeepSeek for English language editing.

## Author contributions

**Conceptualization:** Jie Li, Liang Chen.

**Data curation:** Jie Li, Liang Chen.

**Formal analysis:** Jie Li, Yiming Zeng, Chuang Sun, Yue Guan, Mengmeng Shan, Jinzhou Zhu, Qiying Yao, Liang Chen.

**Funding acquisition:** Mengmeng Shan, Jinzhou Zhu.

**Investigation:** Yiming Zeng, Chuang Sun, Yue Guan, Mengmeng Shan.

**Methodology:** Jie Li, Mengmeng Shan, Jinzhou Zhu, Qiying Yao, Liang Chen.

**Project administration:** Jinzhou Zhu, Liang Chen.

**Software:** Jie Li, Yiming Zeng, Mengmeng Shan, Liang Chen.

**Supervision:** Qiying Yao, Liang Chen.

**Validation:** Jie Li, Yiming Zeng.

**Visualization:** Jie Li, Yiming Zeng, Yue Guan, Qiying Yao, Liang Chen.

**Writing – original draft:** Jie Li, Yiming Zeng.

**Writing – review & editing:** Chuang Sun, Yue Guan, Mengmeng Shan, Jinzhou Zhu, Qiying Yao, Liang Chen.
